# The microbiology of Uganda’s large freshwater lakes experiencing anthropogenic and climatic perturbations: why it matters—a review

**DOI:** 10.1098/rspb.2024.3072

**Published:** 2025-06-11

**Authors:** Daniel Abiriga, Robinson Odong, Grace Kizito Bakyayita, Ronald Semyalo, William Okello, Hans-Peter Grossart

**Affiliations:** ^1^ Department of Environmental Sciences, Kabale University, P.O. Box 317, Kabale, Uganda; ^2^ Department of Zoology, Entomology and Fisheries, Makerere University, P.O. Box 7062, Kampala, Uganda; ^3^ Department of Environmental Science, Kyambogo University, P.O. Box 1, Kyambogo, Uganda; ^4^ National Fisheries Resources Research Institute, P.O. Box 343, Jinja, Uganda; ^5^ Department of Plankton and Microbial Ecology, Leibniz-Institute of Freshwater Ecology and Inland Fisheries, P.O. Box 16775, Stechlin, Germany; ^6^ Institute for Biochemistry and Biology, University of Potsdam, P.O. Box 14469, Potsdam, Germany

**Keywords:** African Great Lakes, antimicrobial resistance, biogeochemical cycling, climate change, freshwater microbial ecology, plastisphere microbes, tropical lake pollution

## Abstract

Intensification of pollution of African water resources due to human activities together with climate change has serious implications for Africa’s blue economy, biodiversity and human health. Despite these overwhelming threats, there is limited research as evidenced by the underrepresentation of Africa-based data in global ecological and biochemical models. This review, therefore, aims to highlight key challenges and existing research gaps, particularly in Ugandan freshwater ecosystems. We focus on lake microbiology as this scientific field has been greatly underrepresented. Aquatic microorganisms are situated at the base of lake food webs and thus play crucial roles in the evolution and maintenance of water quality, attenuation of pollutants, and control of biogeochemical cycling through the microbial loop. Until now, the microbiology of Ugandan lakes has not been systematically studied. Thus, many open fundamental microbial ecology questions need to be urgently addressed to generate valuable information to advance future research, education, management and policy in Uganda and beyond. These include, but are not limited to: identification of microbial taxa and functional genes in relation to anthropogenic and climatic influence; seasonal and spatial variation in species diversity and functions; diversity and functions of planktonic, sediment, biofilm and mat communities; antimicrobial resistance burden; plastisphere communities; and geomicrobiology.

## 1. Introduction

The African continent is privileged to have about 677 lakes [1], of which the seven largest are found in and around the East African Rift Valley, designated as the African Great Lakes (AGLs) [2]. These AGLs are Victoria, Tanganyika, Malawi, Turkana, Albert, Kivu and Edward [3]. Three of these are located in Uganda, although they are transboundary: Lake Victoria, shared with Tanzania and Kenya, and lakes Albert and Edward, both shared with the Democratic Republic of Congo (DRC). The AGLs play important roles in the socio-economic development of riparian countries, through supporting the continent’s blue economy. For example, in Uganda, the fisheries sector supports millions of people through livelihoods and sources of animal protein [4,5]. In the financial year 2021/2022, fisheries and water resources contributed 2 and 2.1%, respectively, of the gross domestic product [6].

The Uganda Government has of late been supporting private investors to set up manufacturing and processing industries in the country to promote import substitution through value addition of locally produced products. Consequently, the number of industries has skyrocketed from about 80 in 1986 to 4920 in 2019 [7], giving rise to regional industrial parks across the country. While this industrialization strategy has contributed to economic development, it has taken place at the expense of the environment. Most of the industries lack proper onsite waste treatment facilities, thus discharging untreated or partially treated effluents into the environment, often into aquatic systems. For example, a survey of 151 industries in 2020 showed 30% compliance with the national wastewater discharge guidelines [8]. Thus, there is ongoing pollution of many aquatic ecosystems, such as lakes, rivers, streams, wetlands and groundwaters [9–13], threatening the country’s blue economy.

Among the many freshwater lakes in Uganda that have been adversely affected by anthropogenic activities, Lake Victoria is threatened by industries, agriculture (tea and sugarcane growing) and catchment deforestation; Lake Kyoga is impacted by industries and agriculture (coffee, food crops and livestock rearing); and lakes Albert, George and Edward are harmed by oil exploration and livestock rearing. In addition, the basins are marked with urban towns and there are cases of other anthropogenic perturbations, such as discharge of raw sewage [14,15] and effluents [12], numerous unregulated small-scale metal works and fabrications, and car garages [9]. As a result, runoff from the catchment transmits complex pollutant cocktails to the lakes, including both legacy contaminants and contaminants of emerging concern (CECs), simultaneously with heavy loads of organic matter (OM). This allochthonous OM may partly stem from natural litter decomposition, driven primarily by microbial communities and detritivore invertebrates [16,17]. Decomposition by the latter group has been found to be strongest in tropical areas [18], but this is a general trend owing to the inherent conducive pH and temperature conditions found in tropical climates [19,20].

Microorganisms play crucial roles in transforming or degrading organic pollutants introduced into lake ecosystems. These microbial processes drive biogeochemical cycling of important nutrients, e.g. C and N, thereby sustaining a bottom-up energy flow [21–23]. A wide range of microorganisms, including oligotrophic and copiotrophic prokaryotes, as well as saprotrophic fungi, are involved. Until now, however, neither microbial communities nor their functional genes in most Ugandan lakes have been studied by using state-of-the-art tools. Fundamental microbial ecology aspects such as which microbial species are present in the lake water and sediment, spatial and seasonal variations as indicated by alpha and beta diversities, community assemblages, taxa co-occurrence, functional genes, phytotoxicity and antimicrobial resistance (AMR) spread remain largely unexplored. Moreover, there are limited comparative studies on the microbiology of these lakes. This is surprising, as many of these lakes differ in abiotic and biotic conditions, which directly influence the microbial community composition and structure. This review discusses the present understanding of the microbial ecology of Ugandan large lakes, identifies the main threats that may accelerate microbial activities, and proposes future research needs to provide new knowledge about microbial ecological processes and mechanisms. While we focus on Ugandan lakes, the issues discussed and the insights and recommendations on microbial ecology studies are broadly applicable to other tropical lakes with even less studied microbiomes [24,25].

Owing to limited studies and hence paucity of information, this review does not discuss microbial communities and bacterial taxa expected in the lakes. Readers interested in the description of microbial taxa of African freshwater lakes can access such information elsewhere [3,22]. The literature focusing on microbiology was retrieved from Google Scholar using the key terms ‘the microbiology or microbial ecology or microbial composition of Uganda lakes or African Great Lakes or the names of the individual lakes Victoria, Albert, Kyoga, Edward, and George’. The top hit papers of up to 15 pages per search were individually scrutinized for suitability, and those falling within one or more of the themes were critically reviewed. These 15 pages were considered because the most relevant papers for this review can be found there. Since this is not a systematic review, we did not focus on the quantitative presentation of the bibliographic details.

## 2. Current knowledge and research gaps on Ugandan large freshwater lakes

**Figure 1.**
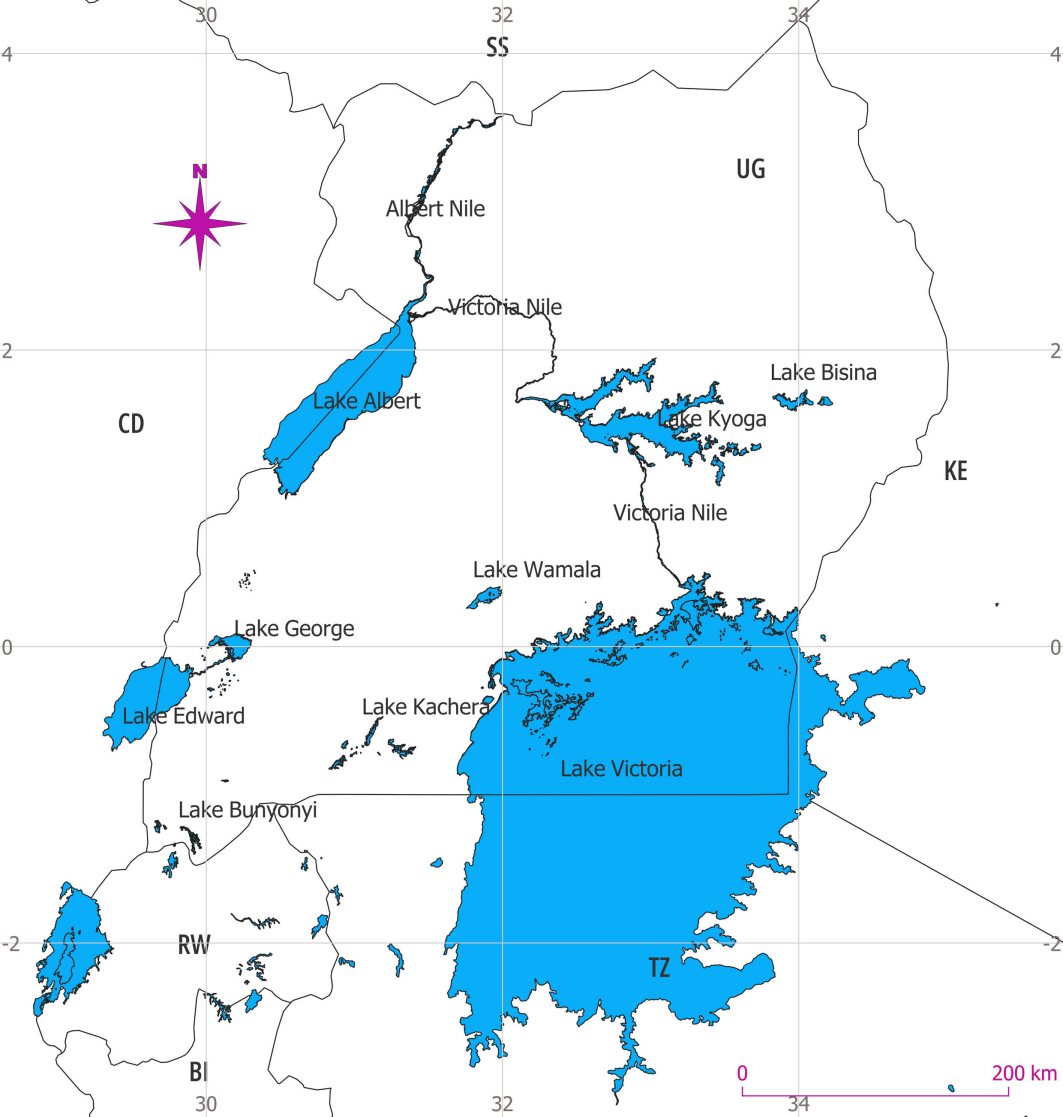
The 10 major lakes of Uganda. Note that Lake Kwania is not labelled as an independent lake, as it is an extension of Lake Kyoga (the topmost arm). The two letter codes represent two-letter country codes: BI, Burundi; CD, Democratic Republic of Congo; KE, Kenya; RW, Rwanda; SS, South Sudan; TZ, Tanzania; UG, Uganda.

There are more than 100 lakes in Uganda [26], distributed across the country, although the majority are located within the central-southern region. The main lakes, in order of decreasing surface area, are Victoria, Albert, Kyoga, Edward, Kwania, Wamala, Bisina, George, Bunyonyi and Kachera [26,27]. In the literature, however, lakes Victoria, Albert, Kyoga, Edward and George (figure 1) feature frequently and are considered the most important water resources in terms of ecosystem service provision and economic potential and are thus the focus of this paper.

### (a) Lake Victoria

Lake Victoria is by far the most studied among all freshwater lakes in Uganda but is still faced with multiple challenges and knowledge gaps. The major land use types in the Lake Victoria basin are urban settlements, industries and agriculture, which have put a lot of pressure on the ecosystem, as evidenced by a drastic decrease in wetland coverage [28] that would otherwise provide natural treatment to laden surface runoff waters ending up in the lake [9]. The continuous degradation of the riparian wetlands means that this ecosystem service is to a large extent altered or even lost, likely increasing the amount of nutrients, OM and CECs in the lake, as well as enhanced greenhouse gas (GHG) emission from the wetlands. This is further exacerbated by a remarkable proliferation of cage fish farming on the lake [29–31], which is associated with negative impacts such as nutrient enrichment, leading to harmful algal blooms (HABs). An evident research gap is to understand how bacterioplankton communities change over time as a function of water quality deterioration. Employing metagenomics to profile microbial genomes for functional traits, metabolism, biogeochemistry and community-scale functional networks can provide valuable data on nutrient cycling. On the other hand, the available data on phytoplankton are inconsistently collected and of low temporal and spatial resolution. This calls for the need to collect high-frequency long-term monitoring data, e.g. by partner states and other collaborative networks such as GELON (https://gleon.org/) and ACARE (https://www.agl-acare.org/).

Lake Victoria receives a discharge of 778.3 m^3^ s^−1^ from 23 rivers [27] in addition to surface runoff from the drainage basin, but has only one outlet, the River Nile. Owing to heavy rainfall in the East African region [13,32], the lake water level rose to a record high on 19 May 2020, reaching up to 13.49 m relative to reference point at Jinja Pier [8]. The incident triggered a risk mitigation measure at Nalubaale Dam to allow more water flow, which caused a cascade of rise in the water levels of the downstream lakes Kyoga and Albert (figure 1 and see text below). From a microbial point of view, such perturbations are likely to impact nutrient and mineral distribution, microbial assembly, microbial turnover of r- and K-strategists, and biodegradation and hence dissolved oxygen (DO) content. Consequently, the research focus should be directed towards biogeochemical processes and notably OM decomposition. This can be achieved, e.g. via profiling the dynamics in microbial community composition and metabolic pathways in the water column and sediment on a long-term basis to assess the recovery and hence resilience of the lake to stochastic climatic events.

Until now, the majority of studies that incorporated microbiology in their investigation have mainly focused on limited aspects such as AMR of *Escherichia coli* isolates [33], prevalence of vibriophage and enterovirus [15,34,35], bacterial contamination of fish [14,36,37], detection of fish viruses [38], archaeal nucleic acid [39], aerobic anoxygenic phototrophs [40] and toxigenic cyanobacteria and their toxins [41–43]. While the studies have contributed to our understanding of microbial life in Lake Victoria, they are narrow in scope and address only specific issues, therefore failing to resolve fundamental ecological questions regarding microbiology in general, e.g. on alpha and beta diversity, lake-wide microbial community composition and dynamics, as well as biogeochemical cycling. Moreover, no study has comprehensively addressed the lake’s microbiology from a multi-taxa, holistic microbial approach encompassing bacteria, archaea, fungi and viruses. Given the roles of these taxa in ecosystem functioning, there is an urgent need to uncover the ‘microbial dark matter’ of Lake Victoria. Despite being the largest of the AGLs by surface area, the microbiome of Lake Victoria has never been characterized by advanced tools like metagenomics. In fact, only three of the AGLs have metagenomic data: Tanganyika [44,45], Kivu [46–48] and Malawi [49].

For a better representative characterization of the lake’s microbiome, sampling sites should be systematically distributed either randomly or in transects from the most polluted nearshore areas (littoral) to the lake’s centre (pelagic). This will put into perspective the influence of anthropogenic disturbance found at the bays relative to open water. Since Lake Victoria stratifies [50] in the dry season and mixes in the wet season [51], it is also critical to consider these changes in a representative study design, as microorganisms show taxon turnover [52] and compositional change in tropical reservoirs [53] in response to hydrodynamic fluctuations.

### (b) Lake Kyoga

Owing to the hydrological connectivity of lakes Victoria and Kyoga through the Victoria Nile, the latter is presented next. Lake Kyoga is the third largest water body in Uganda after Lake Albert. It has a mean depth of just 3 m and a maximum depth of 7 m [26,27]. The lake receives much of its water from the 130 km long Victoria Nile draining Lake Victoria [27]. Consequently, any rise in water level upstream leads to increased volumetric flow, directly affecting the hydrology of Lake Kyoga. Besides the natural phenomena highlighted above, pollution from the increasing number of industries within the drainage basin of Lake Kyoga from the districts of Luwero, Nakasongola and Mbale, together with agricultural practices, especially livestock rearing and rice growing in Lira, Dokolo, Mbale and Nakasongola districts, are major anthropogenic threats to the lake’s ecosystem [54]. While industries impact the lake by discharging toxic industrial wastes, nutrient (C, N) loading, agrochemicals and swamp reclamation are threats from emerging agricultural practices.

Currently, there is limited scientific research on Lake Kyoga, with most of the literature skewed towards fish biology and water chemistry [54–56], with no documentation of microbial ecology-related topics. Hence, microbial research is urgently needed to assess the deterioration in aquatic health under rapidly changing environmental conditions. Such studies should comprise both chemical and microbiological analyses. For instance, the climatic perturbations will destabilize any established microbial assemblages and interactions, and will in addition completely alter water chemistry, e.g. by introducing new ions and elements and by sediment dislodgement, which re-suspends particulate matter-bound pollutants such as nutrients, heavy metals and CECs. Thus, assessing the impact of such climatic perturbations on the dynamics of microbial diversity and species composition, including microbial co-occurrence, will contribute to answering fundamental ecological questions on disturbance–diversity models [57] and on deterministic versus stochastic community assembly [58,59] under field conditions. This can be coupled with a metabolic analysis to decipher impacts on metabolic cycling, e.g. of OM, nutrients and other elements. Owing to the morphometric characteristics of the lake, especially its shallow depth, the sampling design can be rather simple. Hence, it will be interesting to assess the impacts of seasonal changes on the chemical and microbial composition and their impact on water quality and thus human and environmental health.

### (c) Lake Albert

Lake Albert is one of the AGLs located in the East African Rift Valley. It is a transboundary lake shared by Uganda (54%) and DRC (46%) [60,61]. The lake is at the lowest latitudinal point (640 m a.s.l.) [27], making it prone to pronounced flooding. It is connected to Lake Victoria via Lake Kyoga by the Victoria Nile. As such, the rise in the water level of Lake Victoria in 2020 resulted in extensive flooding downstream that displaced the surrounding communities at the lake shores, e.g. residents of Ntoroko and Buliisa Districts [62]. Consequently, the Albert Nile, which forms the White Nile tributary, also caused widespread flooding downstream in the areas of Pakwach, Obongi, Moyo and Adjumani districts. The Lake Albert delta on the north end of the lake created by the Victoria Nile from Murchison Falls is an important ecological site, one of the Ramsar sites in Uganda inhabited by a rich aquatic biodiversity. Similarly, the delta and numerous swamps created by the River Semliki on the southern lakeshore are other important biodiversity hotspots.

The petroleum potential of Uganda was documented in 1925, and the first well was drilled in 1938 in the Albertine Graben [63], with the commercial viability of the oil and gas deposit declared in 2006 [64]. The oil and gas exploration in the Albertine Graben is an anthropogenic activity of serious concern, with the potential to contaminate the entire lake ecosystem [65]. There are five exploration areas located on and around Lake Albert [64]. Any pollution through oil leakage or spillage into such enclosed water bodies will have highly disastrous consequences [2], especially as some of the production sites are located within the body of the lake. Besides these operational risks, inadequate waste management practices can lead to discharge of pollutants such as spent solvent and catalysts, which can potentially pollute the lake with legacy pollutants and CECs. Environmental pollution has already been listed as one of the negative expectations of the oil and gas development in the Albertine region [66].

Microbial studies of Lake Albert are relatively scarce, with the most notable being the comparison of its aerobic anoxygenic phototrophs with those of lakes Victoria, Edward and Kivu and other lakes from Mongolia, Germany and Antarctica [40]. Another relevant study is the characterization of the protistan composition of four East African lakes: Victoria, Albert, Edward and Kivu [67]. Generally, there is very limited scientific information about Lake Albert [65]. The impacts brought about by climatic disturbances on the microbiology of the lake can be examined by replicating the approaches discussed above for Lake Kyoga. Another innovative aspect of microbiological research is a focus on the impacts of oil production on microbial community dynamics, preferably on a long-term basis. More specifically, both lake sediment and water should be sampled to analyse sediment-associated dense non-aqueous-phase liquids and water-associated light non-aqueous-phase liquids together with miscible fractions, respectively. In both sample types, extended chemical analyses should be complemented with that of microbial ecology such that the presence of aromatic-degrading microbes is used as a proxy for oil hydrocarbon pollution of the lake, but will also suggest an ongoing *in situ* bioremediation. Lake morphometric aspects to consider for the study design are distance from bays to offshore sites, depth and seasonality.

Nutrient enrichment, especially at the south and north shores of the lake [65], likely transported by the inflow rivers, holds a high potential for eutrophication and other negative impacts. The flourishing economic activities in the Albertine region are likely to lead to rapid population growth, whose activities, e.g. deforestation, intensive land cultivation and livestock rearing [65], will aggravate the problem of eutrophication. Thus, there is an urgent need for continuous monitoring of the lake, given the current climatic and anthropogenic pressures.

### (d) Lake Edward

Lake Edward is the fourth largest lake by area in Uganda. It is a transboundary water body together with Lake Albert lying in the western arm of the Great East African Rift Valley. It is the fourth major contributor to Uganda’s blue economy [68]. The lake is shared by Uganda (29%) and DRC (71%) [60]. One of the main inflows into the lake is via the Kazinga Channel, which connects lakes George and Edward, while River Semliki forms the main system draining Lake Edward. Scientific information about Lake Edward has been [69], and still continues to be, limited. One of the factors for the lack of extensive scientific studies on the lake is its inaccessibility and insecurity in the area. The ongoing armed conflicts in the eastern DRC remain a big threat, although the Ugandan side is peaceful, and with the discovery of viable oil deposits in the basin, there is a likelihood of increased accessibility to the lake. Already three oil and gas production sites are being developed in the area [64], but this economic activity poses serious environmental challenges. In light of this, there is a need to carry out comprehensive studies in order to guide approaches to protect the lake’s aquatic system. These studies should encompass chemical analyses and microbiological investigations to effectively monitor the effects of the oil and gas industry, as with Lake Albert.

While data on lake chemistry are available (see references in Musinguzi *et al*. [68]), there is a notable scarcity of microbial studies on Lake Edward. The most recent publications on the microbiology of Lake Edward came from the same authors as for Lake Albert [40,67,70]. Studies intended to document the impacts of oil production on microbiology and water quality can be designed as proposed for Lake Albert. A significant portion of Lake Edward is eutrophic, although some areas are mesotrophic [70,71]. The lake is thermally permanently stratified at 70−80 m depth, with the water below this mark being anoxic and nutrient-rich [72]. Such limnological conditions make the lake an ideal ecosystem for exploring various microbial ecology aspects, including light-independent (dark) oxygen production [73,74]. Data on Lake Edward indicate that the lake undergoes seasonal stratification and mixing [70], a phenomenon worth investigating to assess its impact on microbial community assembly, as well as microbial composition as has been previously reported for subtropical and tropical systems [75,76]. When designing sampling for microbial studies, it is essential to consider the morphometric aspect of distance from the bays through littoral to pelagic habitats, as resource availability in these zones significantly influences microbial assembly and composition.

### (e) Lake George

Lake George is entirely located in Uganda. The lake is shallow, having a maximum and a mean depth of 7 and 2.4 m, respectively [26]. A larger proportion of up to three-quarters of the lake basin, including the Kazinga Channel, is located in the protected areas of Queen Elizabeth National Park and its reserves, and its wetlands have been designated a Ramsar Site since 1995 [60,68]. The lake is also located in the Albertine Graben and connected to Lake Edward via the Kazinga Channel. The hydrological connectivity means that there is physical interaction between the two lakes, explaining why in the literature they are often considered together.

The lake is highly productive and characterized by green waters due to high phytoplankton productivity [68], making it hypereutrophic with a dominance of cyanobacteria [70,77]. The chemical characteristics of the lake have been studied in the past [60,68,78], but no literature is available on its microbiology other than phytoplankton and cyanobacteria. Given the roles of other microbial taxa in aquatic ecosystems and their response to the oil and gas development in the basin of Lake George, it is pertinent that microbial ecology should be studied together with chemical analyses. The inherent shallowness of the lake simplifies the required sampling efforts, which can be as those recommended for lakes Albert and Edward.

## 3. Cross-cutting threats: microbial ecological processes

### (a) Biotransformation of pollutants

Previous studies have measured trace metals, xenobiotic organic contaminants (XOCs) and heavy metals in Lake Victoria water and sediment [8,10,79,80]. In view of the various anthropogenic activities discussed for the individual lakes as well as newer emerging contaminants, there is an urgent need for continuous monitoring of XOCs (e.g. from oil production and pesticides) and heavy metals. The monitoring can be done through extended chemical analysis coupled with microbial ecology for different aquatic ecosystems, including wetlands, streams, rivers and lakes. This will enable a better insight into environmental processes, such as biodegradation and biotransformation. For example, detection of methylmercury in sediments would indicate the microbially driven transformation of mercury, which can be confirmed by detecting the catalytic diagnostic microbial enzymes using polymerase chain reaction (PCR). Other elements that undergo similar biochemical transformations are selenium and arsenic [81]. Using a combination of PCR with 16S rDNA metabarcoding will also reveal the microbial taxa involved in the biotransformation. In the case of XOCs, sometimes the intermediate compound formed in the transformation is even more toxic or more persistent than the parent molecule. In addition, their biotransformation often involves novel specialist microbes with unique metabolic repertoires. Hence, future studies on legacy XOCs and heavy metals should involve microbiological studies to comprehensively understand the underlying microbial pollutant transformation processes.

### (b) Biodegradation and biogeochemical cycling

**Figure 2.**
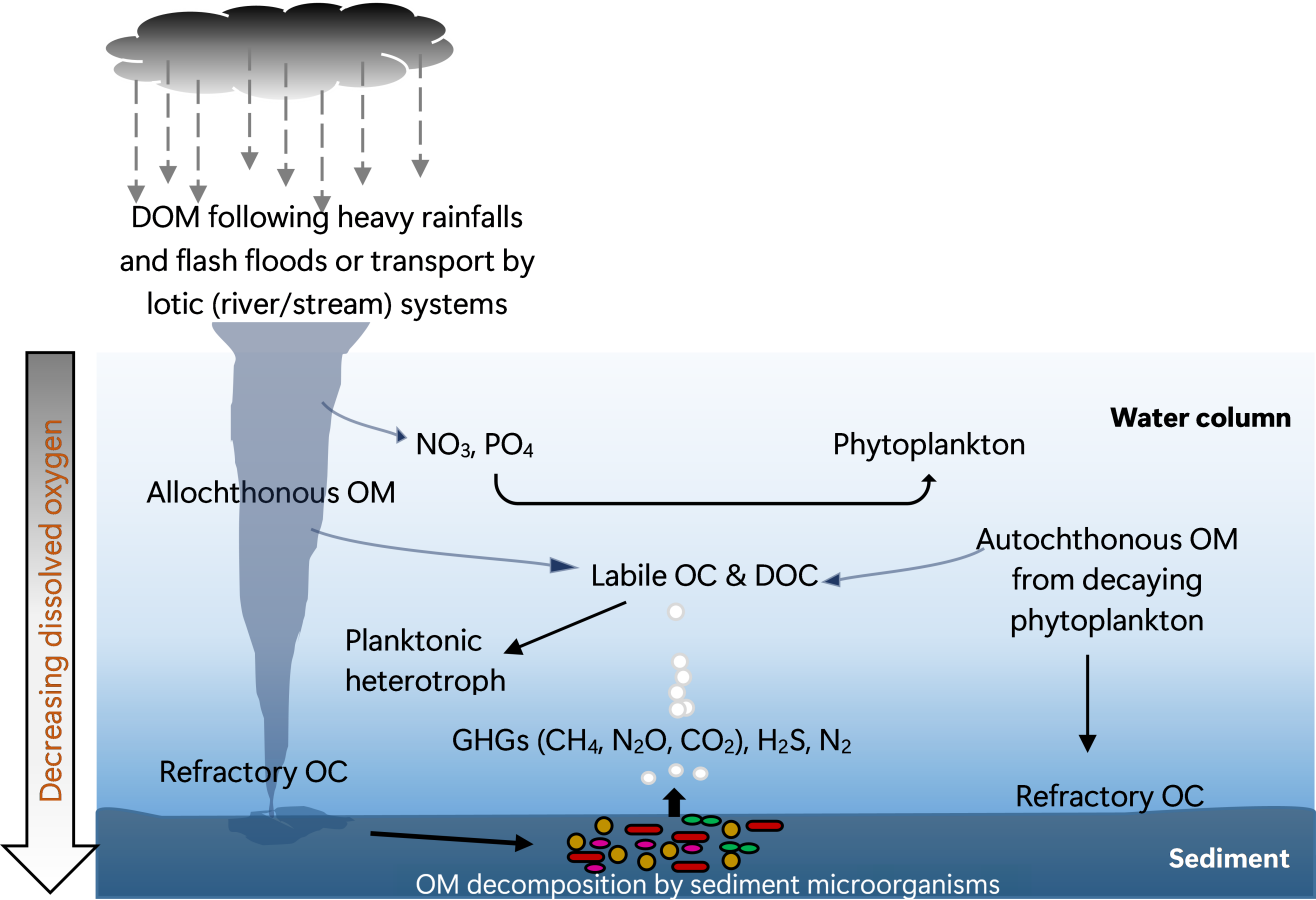
Schematic representation of organic matter (OM) attenuation in a lake experiencing increased OM and nutrient input from terrestrial and river runoff following extreme weather events. DOC, dissolved organic carbon; DOM, dissolved organic matter; GHGs, greenhouse gases; OC, organic carbon.

The organic carbon (OC) transported into lakes may include labile dissolved OC (DOC) and more refractory components. Generally, the labile DOC fractions are readily depleted from the water column [23,51] by planktonic heterotrophic copiotrophs employing the r-strategy mode of growth, resulting in a rapid decrease in DOC levels with depth. Thereby, refractory OC will dominate the bulk of the OM that eventually gets deposited on the lake bottom (figure 2), a substrate that selects for K-strategist microbes. Although this may represent the ideal scenario in modest perturbations, enhanced sedimentation consisting of more labile OC is likely to occur during episodes of intense precipitation, a pattern that recently has become common owing to climate change.

The decomposition of DOC may proceed through microbial activity and photodegradation. The latter pathway requires the upper water layer to be mixed and clear and the photic zone to be of greater depth. In most tropical lakes, however, this is seldom the case. In Lake Victoria, for instance, the water is quite turbid [51], with a large portion of the lake representing the aphotic zone. Thus, microbial OM degradation is expected to be the dominant attenuation pathway. Microbial OM degradation proceeds via either aerobic or anaerobic processes. Aerobic microorganisms utilize oxygen as terminal electron acceptor, a process that depletes DO in the water column, creating a ‘dead zone’ that in turn creates habitat fragmentation [82–84].

In stratified lakes, DO concentration decreases with depth, leading to the eventual development of anoxia at depth. Loss of oxygen in the water column is further exacerbated by hydrodynamic processes such as prolonged and more stable thermal stratification [85]. Hence, in moving towards the bottom, OM degradation proceeds via anaerobic respiration, in which terminal electron acceptors other than oxygen, such as nitrate, manganese(IV), iron(III) and sulfate, are utilized. This produces a number of gaseous by-products, including methane, carbon dioxide, nitrous oxide, hydrogen sulfide and hydrogen, with carbon dioxide, methane and nitrous oxide being the major atmospheric GHGs. As a result, human activities together with climate change will transform the AGLs and their wetland networks more and more into active ecosystems where substantial emissions of GHGs occur. Studying the microbiology of these lakes and the extensive wetland systems, especially the sediments, is of great importance in understanding the biogeochemical cycling, as evidence from tropical and subtropical environments indicates higher species diversity and abundance of methanogens and sulfur metabolizers in the sediment relative to the water column [24,86,87]. This is pertinent since most freshwater ecosystems from the Global South, including the AGLs and their extensive wetlands, remain understudied [19,88,89], especially for GHG emission potential, making them largely underrepresented in global empirical datasets and climate models, resulting in high uncertainty in the regional and global GHG budget estimates [90,91].

## 4. Cross-cutting threats: contaminants of emerging concern

### (a) Plastic pollutants as surfaces for microbes

Plastic pollution is a matter of increasing global concern [92–94]. The lifespan of plastics ranges from a few years to centuries [95], making them persistent environmental threats. Three potential hazards ensue: (1) physical blockage of the gastrointestinal systems of invertebrates and vertebrates, entanglement and entrapment, entry into the food chain or blood circulation and respiratory systems, particularly as micro- and nano-sized plastics [96–99]; (2) leaching of toxic additives such as phthalate and bisphenol A into the environment [96–98]; (3) and transmission of pathogens in the aquatic environment [96,97]. In addition to dispersing microbes in the aquatic system, microbial colonization of plastics may result in depolymerization, as has been observed for hydrolysable plastics, e.g. polyurethane [93]. However, the lack of functional groups in polymers like polyethylene and polypropylene makes them highly recalcitrant with very low biodegradability [93]. Hence, there is a consistent increase in the accumulation of plastics in the environment, and because lakes are usually at the lowest points of landscapes, they receive and accumulate higher amounts of this pollutant, yet its effect on microbial community composition and functioning is not well understood [100,101].

Plastic pollution constitutes a serious problem in Uganda, and attempts to make a total ban on the production and sale of plastic bags, which are the major plastic pollutants, have been unsuccessful owing to lack of political support. Thus, plastics continue to be emitted into the environment at an increasing rate, with the result that, e.g. the major drainage channels discharging water to Lake Victoria are always polluted and even clogged with plastics. Therefore, it is of great importance to conduct research on plastics dispersion and the colonizing microbial communities in the Ugandan lakes. Emphasis can be placed on the microbial diversity, their functional roles, metabolic pathways and enzymes actively expressed.

### (b) Antimicrobial resistance

AMR is an emerging global issue, which is putting a high burden on the health systems, as it is expected to cause more deaths annually [102]. From the viewpoint of an environmental dimension of AMR, the problem is further amplified in the presence of plastic pollution, which has been shown to facilitate horizontal gene transfer, including antibiotic resistance genes (ARGs), between biofilm bacteria on the ever-increasing plastic surfaces [103,104]. Overall, AMR presents a major setback in the struggle for sustainability, explaining why multinational initiatives to fight it have been established, e.g. the Joint Programming Initiative on Antimicrobial Resistance.

Waste streams from livestock farms and effluents from hospitals and municipal wastewater treatment plants (WWTPs) are the main sources of antimicrobial agents in the environment [105–107]. In Uganda, the ever-increasing number of cage fish farms in lakes, inadequate wastewater treatment and selling of antibiotics over-the-counter without prescription foster the spread of AMR in the environment. Hence, there is the likelihood of development and spread of AMR, including multidrug resistance, in Ugandan lakes, as well as among bacterial taxa through horizontal gene transfer involving plasmids, transposons and integrons [108,109]. Already reports are indicating AMR of *E. coli* and *Klebsiella pneumoniae* isolates from Lake Victoria [28]. However, more studies are required to provide a better understanding of the occurrence of residual antibiotics and ARGs in Ugandan surface waters, especially those receiving runoff from animal farms or effluents from hospitals and municipal WWTPs as well as landfill leachate since medical wastes are indiscriminatly disposed of with municipal solid wastes. The effluents and leachate provide a highly dynamic mixture of various pollutants, including ARGs from pathogenic bacteria, high concentrations of OM, nutrients, heavy metals and CECs, providing ideal conditions for promoting and selecting for ARG transfers between human–animal pathogens and environmental bacteria. The resulting health risks for humans and animals remain largely unknown, and studies to better understand the underlying processes and mechanisms require sampling water, particulate OM, plastic debris and sediment [110,111].

### (c) Global warming and aquatic microbes

The East African lakes are potentially very susceptible to climate change [3]. Increase of lake water temperature will cause a shift in the diversity of phytoplankton and surface-dwelling bacterioplankton towards more heat-tolerant species. Furthermore, the phytoplankton assemblages under this condition are expected to be dominated by cyanobacteria [112], some of which are HAB-producing species, e.g. *Microcystis* and *Dolichospermum,* thereby posing serious health risks. This is further exacerbated by nutrient enrichment in lakes stemming from increased runoff due to intense rainfall events of extreme climatic conditions like tropical cyclones and El Niño (Figure 2), promoting bloom-forming cyanobacteria [113], some being capable of fixing nitrogen through diazotrophy and obtaining phosphorus from organic and inorganic sources [112]. Thus, continuous monitoring of water temperature together with microbial analysis will shed light on the impacts of climate change on the Ugandan lakes. The nutrient enrichment in lakes arising from increased runoffs from extreme climatic events will, in addition, promote the development of anoxia in the hypolimnion of stratified lakes through increased consumption of DO via aerobic OM degradation, a condition that allows specific microbial communities and processes to occur, including GHG evolution. Moreover, rise in lake water temperature decreases the dissolution of oxygen, affecting the replenishment of DO in lakes. Another effect of global warming is strengthening lake stratification, which restricts nutrient fluxes from the hypolimnion to the epilimnion [114,115], creating unique microbial communities per layer, as observed for instance in Lake Kivu [46].

On the surrounding wetland systems, extreme rainfalls will likely increase GHG emissions, as intense rainfall causes extensive flooding of wetlands, while receding of flood water exposes sequestered carbon and nitrogen to microorganisms, a scenario associated with enhanced GHG emission [116]. On the other hand, warming of wetlands due to high temperatures will increase the rate of GHG emission, thereby sustaining a positive climate feedback [117].

## 5. Concluding remarks

Despite the societal importance of freshwater lakes, such as nutrient cycling, water provision, fishing and the blue economy, as discussed in this paper, the roles of microbial communities in sustaining the continued exploitation of these services remain less understood in tropical settings. To stress this significance, microbiological studies will generate data on several key issues, such as safety of drinking water as some lakeshore communities use the lake waters with minor treatment, risk assessment of phyto(cyano)toxins (produced by HABs) to fisheries and human health, multidrug-resistant microbes and genes threatening aquatic and human lives, GHG emission and carbon sequestration offsets, and bioremediation potential of microbes and hence resilience to pollution and climatic shocks. Consequently, a multidisciplinary research agenda is needed as many threats are obvious, but their ecological consequences remain largely unknown. To generate in-depth data of enduring quality that drives decision-making, there is a need to involve stakeholders such as the Lake Victoria Fisheries Organisation, Lakes Edward and Albert Fisheries and Aquaculture Organisation, line ministries, and local communities for traditional knowledge in co-creation and design of participatory research. While this will further lead to capacity building, shared responsibility and project acceptance, it may cause delays in implementation owing to competing interests, power imbalance and bureaucracy.

Advanced microbial studies may involve metagenomics or metabarcoding of microbial taxa, including bacteria, archaea, microeukaryotes (protists and fungi) and viruses, e.g. bacteriophages. For the five lake systems considered in this review, a comparative microbial study will shed light on the long-standing microbial ecological concept of biogeography versus ‘everything is everywhere' [118] and keystone taxa. This comparison is intriguing for interconnected versus isolated lakes, which will bring to light the concept of dispersal limitation and homogenizing dispersal as used in mechanistic distinction of stochastic versus deterministic processes. The microbiological studies can be framed around the major themes discussed in this review: biotransformation of pollutants; biodegradation and biogeochemical cycling; plastic pollution; AMR; and impacts of global warming.

From a bigger intra- and inter-continental viewpoint, more research efforts are needed by research organizations and networks located in the AGLs region and partners outside the continent. Comparison of microbial diversity across African lakes and the comparison of tropical versus temperate lakes in terms of microbial diversity and composition through meta-analyses are of primary interest. Currently, the available microbial data from the AGLs stem from a handful of water bodies, namely, lakes Tanganyika, Malawi and Kivu. Moreover, these studies are not led by scientists based in Africa [119], in addition to being infrequent and thus lacking the required detailed temporal and spatial resolutions. Therefore, there is a great need to refocus the global North–South collaborative partnership from the present collect-data-and-publish to institutional capacity building. This will ensure that African-based scientists can lead these research initiatives and be able to secure sustained funding to advance knowledge to transform societies and policies for sustainable development and ecosystem conservation in Africa.

## Data Availability

This is a review paper and therefore does not contain any additional data.
